# Supplementary material for: Are carbon emissions trading and green financial instruments synergistic? -Comprehensive quantitative research based on content analysis
Source: PLoS One. 2024 Mar 7;19(3):e0298601. doi: 10.1371/journal.pone.0298601 (PMC10919601; doi:10.1371/journal.pone.0298601)
Supplement: S1 Data — (ZIP) [file pone.0298601.s001.zip › Data/地级市Z_F工作报告.zip!/╣π╬≈/╣≤╕█╩╨/└┤╫╘╣1⁄2╓┌║┼í╛┬φ┐╦╩2╛▌═°í┐_2017.docx]

各位代表：

现在，我代表市人民政府向大会报告工作，请予审议，并请市政协各位委员和列席人员提出意见。

一、2016年工作回顾

2016年，在自治区党委、政府和市委的坚强领导下，在市人大及其常委会的法律监督、工作监督和市政协的民主监督下，我们高举中国特色社会主义伟大旗帜，坚持以马克思列宁主义、毛泽东思想、邓小平理论、“三个代表”重要思想、科学发展观为指导，全面贯彻党的十八大和十八届三中、四中、五中、六中全会精神，深入贯彻习近平总书记系列重要讲话精神和治国理政新理念新思想新战略，按照自治区“四大战略”和“三大攻坚战”的决策部署，围绕加快建成西江流域核心港口和新兴工业城市目标，大力实施“工业兴市、工业强市，改革引领、创新驱动，东进融合、开放带动”三大战略，坚决打赢“贵港经济翻身仗、城市提升主动仗、脱贫摘帽攻坚仗、作风建设持久仗”四场硬仗，打造“实力、活力、魅力、给力”四力新贵港，努力实现“工业大发展、项目大兑现、服务大提速、城市大变化、县域经济大突破、脱贫攻坚大成就”六大目标要求，全面开展工业提质年、项目落地年、企业服务年、城市建设管理年、县域经济突破年、精准脱贫攻坚年“六个年”活动，经济社会保持平稳健康发展，实现“十三五”的良好开局。我们坚决贯彻落实中央和自治区政策措施，执行稳增长措施有力，特别是招商引资和民间投资工作突出，得到自治区通报激励。预计地区生产总值958.8亿元，增长7.9%；财政收入78.9亿元，增长8.5%；固定资产投资841.7亿元，增长22%；居民人均可支配收入18653元，增长8.5%；其他主要经济指标保持较快增长。

（一）经济发展迈上新台阶

工业增量提质。深入开展“工业提质年”“企业服务年”活动，预计规模以上工业总产值985.7亿元，增长13.3%；规模以上工业增加值299.4亿元，增长8.8%；新增规模以上工业企业31家、总数达430家，产值超亿元工业企业达215家。新兴产业实现重大突破，华奥新能源大客车下线，久久星等54家新能源电动车项目投产，中国—东盟新能源电动车生产基地初具规模，赛尔康电子项目新厂房主体工程封顶，创造了工业项目建设的“贵港速度”，开启了“贵港制造”新纪元。新能源汽车、电子信息、生物制药成为贵港工业的“新三篇”。传统产业提档升级，林板纸、机械制造产业产值首次超100亿元，建材业产值突破200亿元。园区经济持续壮大，园区规模以上工业总产值增长13.2%。持续开展“市长服务企业接待日”活动，解决了235个企业336个问题。

农业现代化步伐加快。预计农林牧渔业总产值331.4亿元，增长4%。粮食产量155万吨，继续保持增产。建设现代特色农业示范区66个，新增港南区四季花田休闲农业（核心）示范区、桂平市粮食产业（核心）示范区等自治区级示范区。落实“双高”糖料蔗基地5.3万亩。建立富硒农产品生产基地62个，获自治区富硒农产品认证26个、中国名优（特色）硒产品8个，形成了“中国硒港”的价值品牌。新增市级以上农业龙头企业18家、农民专业合作社1577家，新建规模养殖场（区）190多个。主要农作物耕种收综合机械化水平达55.8%。

服务业繁荣发展。预计社会消费品零售总额431.9亿元，增长11%。上亿国际汽贸博览城和美家居国际博览城基本建成，中心城区新建新华农贸市场和东湖农贸市场，200多个“电子商务进农村”标准化服务点投入运营。港口货物吞吐量5763万吨，增长8%；集装箱吞吐量16.7万标准箱，增长33%；广西西江航运交易所投入运营，常年开通直达香港货物航班。旅游接待总人数增长15%，旅游总消费增长28%，覃塘区被认定为“全国休闲农业和乡村旅游示范县”，荷美覃塘、四季花田评为广西星级乡村旅游区，举办了中国健身名山登山赛。会展经济取得初步成效, 举办了富硒农产品交易会、第六届广西发明创造成果展览交易会、中国（贵港）木材加工产业发展高峰论坛。金融机构人民币存款余额增长12%，贷款余额增长14.6%。餐饮住宿、保险、快递等其他服务业稳步发展。

项目建设力度加大。深入开展“项目落地年”活动，全市主要建设项目551个，完成投资269亿元，77个项目实现竣工，94个项目实现开工。全力破解瓶颈制约，获新增建设用地13666亩，增长93.4%。储备“十三五”建设项目1367个，总投资5014亿元，其中PPP项目31个，总投资381.4亿元。

县域经济发展壮大。深入开展“县域经济突破年”活动，平南县、港北区、港南区、覃塘区GDP增速和平南县、港北区、港南区财政收入增速高于全区平均水平，五个县市区固定资产投资、居民人均可支配收入增速高于全区平均水平。平南县抓招商、促项目，规模以上工业保持高位增长，得到自治区通报激励；桂平工业持续壮大，规模以上工业总产值突破350亿元；港北区电动车产业发展迅速，产值突破10亿元；港南区林产品加工业提档升级加快，产值达65亿元；覃塘区汽车产业初步成形，广西第二个汽车（新能源）生产基地建设迈出坚实步伐。

科技创新能力不断增强。新认定国家高新技术企业7家，新增自治区级科技创新平台4家，完成科技成果转化12项，每万人口发明专利拥有量增长49.4%。深入实施质量强市战略，新增广西优质工程6个、广西名牌4个、国家质量标杆企业1个。挂牌成立华奥汽车—上海同捷联合汽车工程研究院。

（二）改革开放亮点纷呈

重点领域改革持续深化。加快推进行政审批制度改革，强化行政审批串联转并联、前置转后审、零散转集中 “三个转变”，在国土、住建、环保部门试点“说不”报告制度，行政审批提速87%；清理取消行政审批事项29项，调整17项，清理规范行政审批中介服务事项98项；商事制度改革成效明显，新发展市场主体2.8万户，增长27.3%。深化财政管理体制改革，盘活财政存量资金10.4亿元；落实“营改增”政策，停征或取消行政事业性收费35项，为企业减负1.6亿元。推进国有企业、农村土地确权登记、不动产登记、医药卫生、国有林场、林权、水利、教育、机关事业单位养老保险制度和党政机关公车改革。供销合作社综合改革成为全区典型。

开放合作成效显著。全市招商引资新签约项目394个，合同投资额883.9亿元，其中亿元以上项目224个。铁牛汽车、赛尔康电子、贝丰电子、海南新世通制药等一批新兴产业项目落户贵港，汽车、电子信息产业集群初见雏形。招商引资项目新开工238个，到位资金96.9亿元。外贸进出口总额19.4亿元，贵港港口岸扩大开放项目列入国家口岸发展“十三五”规划，贵港西江保税物流中心（B型）列入自治区规划。成功举办贵港市成立20周年庆祝活动，对外展示贵港开放发展新面貌；加深了与韩国首尔特别市九老区、菲律宾三宝颜市交流合作，与泰国洛坤府建立了友好关系。

（三）城乡面貌日新月异

城镇建设力度加大。深入开展“城市建设管理年”活动，罗泊湾大桥顺利建成，中心城区东环路、贵港二线船闸公路改线枢纽大桥通车，打通6条城区“断头路”，完成中心城区9条主干道“白改黑”工程。青云大桥、解放路和同济大道下穿铁路工程、高铁站前广场、园博园等项目建设加快。推进城市绿化亮化，主要街道四季有花开。创新微信城管、微信政务，积极创建全国文明城市，开展市容市貌综合整治，人居环境持续改善。启动创建桂平市文化艺术城市，平南县滨江亮化工程得到自治区肯定。基本完成东龙镇自治区百镇建设示范镇创建任务和马练瑶族乡少数民族示范项目。全市城镇化率达47%。

基础设施逐步完善。新增等级公路233公里，农村公路522公里，全市所有行政村通达水泥路或沥青路。除险加固病险水库5座，治理中小河流23公里，完成渠道防渗609公里，新增、恢复和改善灌溉面积34万亩。城乡电网供电能力全面提升。

生态环境持续改善。预计单位地区生产总值能耗下降2.5%，规模以上万元工业增加值能耗下降3.6%，全面完成自治区下达的主要污染物减排目标任务。城市环境空气质量优良率92.6%，PM10平均下降14.1%。城市集中式饮用水水源地水质达标率100%。“美丽贵港·生态乡村”活动扎实开展，建成饮水净化示范项目20个，硬化村屯道路330公里，完成自治区示范村屯绿化233个，港南区湛江镇平江村评为“全国美丽宜居村庄示范”；开工建设乡镇污水处理设施10个，新建乡镇生活垃圾转运站21个，实现所有乡镇有垃圾转运站；建立高架网床生态化养殖示范点11个。推进国家森林城市创建，全市森林覆盖率达46.33%。

（四）脱贫攻坚取得明显成效

深入开展“精准脱贫攻坚年”活动，94个贫困村、8万农村贫困人口实现脱贫销号。开展“1+N”产业扶贫，创新推广“一十百千万”产业扶贫模式，2016年脱贫摘帽村均有1个以上产业。开发乡村公益性岗位，转移就业贫困劳动力1630人；发放扶贫小额贷款11.4亿元，带动2.3万贫困户增收。建成贫困村村级公共服务中心63个；补助参加新农合贫困群众18.6万人，提高贫困群众住院报销比例5个百分点；发放2.9亿元资助贫困学生8.7万人；8.6万农村贫困群众纳入低保范围。修建贫困村屯道路1303公里，实施农村饮水安全巩固提升工程31个，改善5623户贫困户住房条件，搬迁贫困群众1018人。

（五）民生事业有新提升

坚持把改善人民生活作为重中之重，财政支出的81.5%用于民生领域，全力推进为民办实事项目。

教育事业加快发展。投入18.8亿元新建和改造校舍93.3万平方米，新建、改扩建幼儿园60所，完成市达开高中扩建一期工程，新建龚州中学、金港小学、港宁小学等学校，启动贵港高中新校区和西江职教园区建设。义务教育巩固率位居全区前茅，学前三年毛入园率比上年提高7个百分点，每万人口高中在校生人数排全区第1位，高考成绩持续攀高。覃塘区通过国家义务教育发展基本均衡县评估验收，港北区、港南区通过自治区义务教育均衡发展督导评估。职业教育、民办教育、特殊教育、民族教育等各类教育成效明显。

文化体育事业繁荣发展。新建广播电视乡镇无线发射站5座、村级公共服务中心80个，完成金田起义遗址博物馆主体工程，开工建设平南县文化体育中心，群众文化活动深入开展；维修保护贵港南江古码头、桂平东塔等自治区级文物；建成4个自治区级非遗传承基地，新增2个自治区级非遗项目。建成市体育中心，成功举办了市第一届体育运动会。

卫生计生工作得到加强。建设港北区人民医院内科楼、平南县人民医院外科楼等8个医院项目。新农合参保率达99.1%。桂平市荣获“国家妇幼健康优质服务示范县”，覃塘区荣获“全国计划生育优质服务先进单位”。

就业形势保持稳定。全市城镇新增就业1.4万人，农村劳动力转移就业新增3.9万人，城镇登记失业率1.1%。

社会保障水平进一步提升。城乡居民社会养老保险参保率达96.7%，城市社区居家养老服务覆盖率达61.3%。投入100多万元改善农村福利机构食堂条件。建设保障性住房3440套，完成货币化安置1109套。实施水库移民项目222个，受益移民6万多人。建成市儿童福利院和市老年人活动中心。

其他各项事业协调发展。加强矛盾纠纷排查化解，群众诉求表达渠道更加畅通。推进公共法律服务体系建设，全市65%的村（社区）建立公共法律服务中心（站、点）。应急处置机制更加完善，妥善处置各类突发事件。国防教育、国防后备力量建设成效明显，荣获“全国双拥模范城”八连冠、“全区双拥模范城”九连冠。加强安全生产工作，安全生产形势总体稳定。“平安贵港”建设有效推进，社会更加和谐稳定。妇女、儿童、残疾人和老年人权益得到有效保障，民族、宗教、侨务和对台工作取得新成效，档案、地方志、社会科学等事业取得新成绩，防震减灾、气象服务、人防建设得到加强，统计、绩效、物价、公积金、机关后勤、二轻联社等工作取得新进步，工会、共青团、妇联、科协、社科联等组织在促进经济社会发展中发挥了积极作用，海关、海事、检验检疫、边检、武警、消防、农垦、邮政、通信、水文、石油化工、烟草、盐业、船舶检验、地质勘探、无线电监测等中区直部门为经济社会发展提供了强有力支持。

（六）政府建设展现新气象

加强政府系统党建工作。认真开展“两学一做”学习教育，政府系统党组织凝聚力战斗力不断增强。按照市委部署开展解放思想、转变作风活动，开展机关“转作风、强责任、优服务、促规范”活动，大力弘扬敢于担当、勇于负责精神，大力倡导“四破四立”“四扬四弃”，以作风的大转变推动干事创业风气的大转变，干部执行力、协同力、创新力进一步增强。

认真执行市人大及其常委会决议和决定，自觉接受市人大及其常委会的法律监督、工作监督和市政协的民主监督，以及社会和舆论监督。坚持重大事项向市人大常委会报告、向市政协通报，认真听取各民主党派、工商联、无党派人士和各人民团体的意见建议，人大代表建议和政协提案办结率100%。

推进政府信息公开，乡村民生项目和资金使用情况实现网上公开。积极回应群众关切，政府热线受理中心及微信、市政微信、政务微信、网络问政平台等常态化政民互动渠道更加畅通。

法治政府建设得到加强，公开部门权力清单和责任清单，严格规范行政执法行为，权力运行更加规范。健全依法决策机制，落实“三重一大”民主决策制度。全面实行政府法律顾问制度。

认真贯彻落实中央“八项规定”精神，坚决反对“四风”，严格执行国务院“约法三章”，“三公”经费实现零增长。强化审计和监察工作，加大扶贫领域监督执纪问责，严肃查处发生在群众身边的“四风”和腐败问题。

各位代表！过去一年的成绩来之不易。这是在自治区党委、政府和市委的正确领导下，在市人大及其常委会的法律监督、工作监督和市政协的民主监督下，在市中级人民法院和市人民检察院的司法保障下，各级各部门和各族人民团结奋斗的结果。在此，我代表市人民政府，向全市各族人民，向人大代表、政协委员，向人民法院和人民检察院，向各民主党派、工商联、无党派人士、各人民团体，向中区直单位和驻贵人民解放军、武警部队官兵，向所有关心支持贵港发展的社会各界人士，致以崇高的敬意和衷心的感谢！

我们也清醒地看到，发展中还存在不少困难和问题，全市综合经济实力还不够强，总量不大，经济增长方式还比较粗放，工业短板仍比较突出，开放合作的广度和深度不够，基本公共服务发展还比较滞后，社会保障体系仍不完善，脱贫攻坚任务艰巨。干部作风还有待进一步转变，政府系统党建工作仍需进一步加强。对此，我们一定高度重视，采取更加有力措施，精准发力、精准施策，积极化解难题，推动经济社会跨越发展。

二、2017年工作安排

今年是实施“十三五”规划的重要一年。自治区第十一次党代会提出营造“三大生态”、实现“两个建成”，谱写建党百年广西发展新篇章；市第五次党代会号召全市上下决胜小康，再创辉煌，全力打造实力活力魅力给力新贵港。我们要按照自治区党委、政府和市委的部署要求，切实把各项工作落到实处。

今年政府工作的总体要求是：全面贯彻党的十八大和十八届三中、四中、五中、六中全会精神和中央经济工作会议精神，深入贯彻习近平总书记系列重要讲话精神和治国理政新理念新思想新战略，统筹推进“五位一体”总体布局，协调推进“四个全面”战略布局，按照自治区第十一次党代会和全区经济工作会议部署，围绕市第五次党代会提出的“13446”工作思路，坚持“稳中求好、稳中快进”的发展主基调，更加注重经济结构调整和动力转换，更加注重质量与速度良好互动，更加注重改革开放和创新驱动，更加注重城乡统筹和协调发展，更加注重保障和改善民生，更加注重风险防范和管控，深化拓展“工业提质年”“项目落地年”“企业服务年”“城市建设管理年”“县域经济突破年”“精准脱贫攻坚年”活动，推动全市经济实现平稳快速发展和社会和谐稳定，以优异成绩迎接党的十九大胜利召开。

今年经济和社会发展的主要预期目标是：地区生产总值增长8%，财政收入增长9%，规模以上工业增加值增长9.2%，固定资产投资增长15%，社会消费品零售总额增长8%，外贸进出口总值增长10%，居民人均可支配收入增长8%，节能减排控制在自治区下达的目标内，居民消费价格指数涨幅控制在3%左右，城镇登记失业率控制在4.3%以内，城镇新增就业1.3万人以上，人口自然增长率控制在11‰以内，常住人口城镇化率达48 %。

为实现上述目标，我们将重点做好以下几方面：

（一）加快转型升级，着力提升经济发展质量

巩固工业主导地位。深化拓展“工业提质年”“企业服务年”活动，年内规模以上工业总产值增长14%以上，培育新增规模以上工业企业30家。支持华奥、铁牛、久久星等企业建设和发展，夯实广西第二个汽车生产基地基础；发展电动车产业，做大做强中国—东盟新能源电动车生产基地。培育发展电子信息、生物制药等新兴产业，力促赛尔康电子、贝丰电子、石家庄药业等项目建成投产，开工建设海南新世通制药、嘉龙海杰电子等项目。提档升级建材、冶金、糖纸、林产品加工、羽绒等传统产业，培育和发展10亿元以上企业。实施产业园区基础设施建设大会战，年内园区新入驻企业50家以上，规模以上工业总产值占全市65%以上。

打造现代特色农业。保障粮食安全，粮食总产量保持在150万吨以上。推进特色农业示范区创建，年内基本建成66个现代特色农业示范区。打造广西乃至全国具有影响力的“中国硒港”，年内建立富硒农产品生产基地5万亩以上，创建富硒农产品品牌5个以上。规划建设10万亩“双高”糖料蔗基地。启动实施20万亩百香果产业三年行动计划和水果产业提质增效行动，年内特色水果突破25万吨。规范发展养殖业，年内30%的规模养殖场达到生态养殖标准。培育新型农业经营主体，年内新增农业龙头企业10家以上，农民专业合作社200家以上，家庭农场100家以上。主要农作物耕种收综合机械化水平达57%以上。

发展壮大服务业。打造高效便捷的区域物流服务平台，规划建设贵港综合物流园，开工建设贵港西江保税物流中心（B型）、林产品加工商贸物流园；加快农贸市场升级改造，在中心城区新建2个农贸市场。构建农村现代化流通体系，创建农产品交易电子商务平台，打造一批国家级、自治区级电子商务示范镇、示范村。发展会展经济，策划举办富硒产品交易会、林产品交易会、汽车展销会、电商节等促销活动。加快旅游业发展，年内接待游客人数增长10%以上，旅游总消费增长15%以上。推进铜鼓湾温泉度假区、太平天国金田起义遗址红色旅游经典景区、西山泉国际养生旅游文化综合区等重大旅游项目建设。加快桂平西山风景名胜区国家5A级景区和平天山国家森林公园、北帝山生态旅游区国家4A级景区创建工作。推进桂平市创建广西特色旅游名县。推动乡村旅游提档升级，创建星级乡村旅游区、农家乐8家以上。培育发展科技服务、信息服务等新兴服务业。

增强县域经济实力。深化拓展“县域经济突破年”活动，实施“五域联动”，推动五个县市区齐头并进、联动发展。年内五个县市区地区生产总值、财政收入增速高于全区平均水平，2-3个县市区地区生产总值突破300亿元，县域财政收入总量占全市财政收入75%以上。引导支持各县市区培育优势主导产业，年内五个县市区规模以上工业增加值增长15%以上，推动桂平市陶瓷业、平南县建材业、港北区电动车产业、港南区林产品和羽绒加工业、覃塘区新能源汽车产业做大做强。推动县域投资上项目，年内五个县市区固定资产投资增长20%以上，2-3个县市区投资总量突破250亿元。实施城乡居民收入倍增计划，年内五个县市区居民人均可支配收入均突破20000元。

（二）深化改革开放，不断增添发展动力活力

强化改革创新。深化财政管理体制改革，加快PPP项目落地，设立政府投资引导基金，全面落实减税降费政策，推进政府购买服务、预算绩效管理。推进审批制度改革，简化审批环节，提高审批效率，推动审批大提速。深化商事制度改革，积极开展个体工商户简易注销登记改革试点。加快国有企业改革重组，加强国有资产监管，增强国有企业发展活力。深化园区管理体制改革。全面启动公立医院综合改革。推行河长制，加强河湖管理。改革城市执法体制、供销合作、农村金融、农村土地、国有林场、水利体制等。实施创新驱动发展战略，年内完成科技成果转化项目8个、高新技术企业数量增长10%以上、每万人口发明专利拥有量增长35%以上；实施科技成果转化大行动、高新技术企业倍增计划、发明专利双倍增计划，设立创新发展基金，建立贵港高新技术产业开发区和一批产业工程研究院。建设中国—东盟新能源汽车生产基地检验检测认证中心，年内打造广西著名商标10个、名牌产品6个。

推进招商引资上项目。深化拓展“项目落地年”活动，切实提高项目落地率、投产率和贡献率，重点破解项目用地和园区基础设施瓶颈制约。持续开展“大招商、招大商”活动，年内引进项目300个以上，合同投资额力争突破800亿元。围绕新能源汽车、电动车、电子信息、装备制造、生物制药和食品加工、林产品加工等产业开展产业链招商，重点打造新能源汽车、电子信息、生物制药等产业集群。推进重点区域招商，面向珠三角、长三角和其他发达地区，瞄准国内外500强、行业100强、央企、大型民企，力争引进一批大项目、好项目。

扩大对外交流与合作。实施加工贸易倍增计划，培育引进加工贸易龙头企业，扩大新能源汽车、电动车、电子信息、芒编工艺品、林产品、农副产品、圣诞节用品等出口。启动口岸开放建设，建成贵港电子口岸，申报进境粮食指定口岸。提高通关便利化水平，整合优化海关、检验检疫等查验部门作业流程。以东盟国家为重点，扩大对外友好交往，争取与一个东盟国家城市建立友好城市关系。

（三）完善基础设施，深入推进城乡统筹发展

推进城市面貌大改观。深化拓展“城市建设管理年”活动，坚持港产城互动融合发展，开展城市总体规划修编；规划建设城市新区，打造集生产、服务和消费于一体的城市综合体；加强城市新区、产业园区功能组合；做好市政设施与园区设施衔接，建设具有城市综合服务功能的产业新城。实施基础设施提升工程，完善中心城区基础设施，建成青云大桥、江南大道、解放路和同济大道下穿铁路工程、贵港至覃塘一级路等城市路桥，打通迎宾大道东侧支路、金田路等“断头路”，完成城区主要道路“白改黑”工程，开工建设园博园、兴六高速公路至城区一级连线提升工程、郁江两岸综合整治工程、鲤鱼江湿地公园、南山片区水环境综合整治工程、港区大道、同济大道、城市地下综合管廊等项目，积极筹备江南大桥、城东大桥、城区第二水源等项目建设。完成城区出入口、街道、公园景区绿化亮化彩化景观改造。编制海绵城市规划，加快建设海绵城市，解决城区防洪排涝问题。打造绿色公交，年内投入新能源公共汽车300辆。鼓励绿色出行，规划建设城市公共自行车系统。加快旧城改造，改善人居环境。深入开展城市管理大行动，继续推进文明城市创建，开展市容市貌专项整治，让城市“更净、更畅、更美、更亮”。开展打击“双违”大会战，坚决遏制乱搭乱建和违规抢建行为。

加快城镇建设。推进桂平市城区堤路园、跨江大桥、污垃处理设施建设，加快平南县城区路桥、公园、滨水特色景观带、农贸市场建设。培育发展特色小城镇，力争将桥圩、木乐、大安、马练列入2017年国家级特色小城镇试点，平山列入自治区级特色小城镇试点，扶持发展大圩、庆丰、石卡、东龙、石龙、金田、镇隆等重点镇。做好农业转移人口市民化工作。

改善农村生产生活条件。全面开展“宜居乡村”活动，巩固提升“清洁乡村”和“生态乡村”活动成果。完善农村公路网络，年内修建农村公路500公里以上。开展水利项目建设大会战，年内实施中小河流治理项目5个，完成达开、六陈、武思江等3个大型灌区续建配套与节水改造，恢复或改善灌溉面积5万亩。加速移民工程建设，完成水库移民新村项目80个以上。

完善交通基础设施。加快路网提级改造，力促贵港至合浦、柳州至梧州高速公路和来宾寺山至贵港、木格至博白水鸣二级公路建成通车；开工建设西外环高速公路，推进贵港至隆安、荔浦至玉林高速公路及梧州蒙山至平南国安、平南四灵至兴业高峰、兴业高峰至大岭、上渡至武林二级公路建设。建设区域性航运枢纽，年内新增2000吨级泊位13个，港口货物吞吐量增长5%以上。加快大藤峡水利枢纽、贵港航运枢纽二线船闸、苏湾作业区一期等项目建设，规划建设贵港至北海城际铁路及中心港区至桂平、平南港区沿江货运铁路和一级公路。坚持铁路、水路、公路、航空、管道建设齐头并进，打通各交通运输体系间的“毛细血管”，实现智能高效、无缝衔接，加快构建四通八达、安全快捷现代综合交通运输体系和现代物流体系。

（四）建设生态文明，大力营造优美宜居环境

强化节能减排。年内单位地区生产总值能耗下降3.5%，规模以上万元工业增加值能耗下降3.6%。严格项目准入，坚决控制新上高耗能、高污染、高排放项目。实施节能降耗重点工程，狠抓火电厂等行业企业超低排放改造。实行最严格水资源管理制度，提高水资源利用率。

推进生态项目建设。积极创建国家森林城市，年内完成植树造林10万亩以上，森林覆盖率达46.41%。启动城区水环境整治，推进水生态文明城市自治区级试点工作。开工建设城东污水处理厂，年内城区污水处理率达到92%以上。开工建设桂平和平南垃圾焚烧发电厂，建成城镇污水垃圾处理设施项目14个。开工建设现代化病死禽畜无害化处理中心。严格落实永久基本农田红线管控，盘活存量建设用地，依法处置闲置土地，节约集约利用土地。

加强污染防治。强化扬尘治理，重点开展燃煤小锅炉和木材加工企业的环境整治，坚决完成上级下达的城市环境空气质量优良天数目标任务和PM10指标任务。推进水土污染防治，城市集中式饮用水水源地水质达标率和地表水国控断面水质优良比例达到自治区考核要求。认真落实环境保护“党政同责、一岗双责”，实行环境监管网格化管理，依法惩治环境违法行为。

（五）强化精准脱贫，全力打赢脱贫攻坚战

深化拓展“精准脱贫攻坚年”活动，年内78个贫困村、5.4万农村贫困人口脱贫销号。

千方百计促进贫困群众增收。年内建成20个特色产业脱贫示范基地，扶持5个贫困村开发旅游项目，确保每个县市区有2个以上特色产业、每个贫困户有1个以上特色产业；引进民间资本参与产业扶贫开发，确保2017年预脱贫的每个贫困村至少有1个农民合作社；大力支持贫困村发展集体经济。继续推动扶贫小额信贷、农业保险等金融扶贫。加快贫困村电商服务点建设，实现电子商务服务点2017年预脱贫村全覆盖。加强农村贫困劳动力职业技能培训，年内转移贫困人口就业1500人以上。

加快补齐贫困村基础设施短板。完善贫困村路网，年内建成贫困村屯道路900公里以上。实施农村饮水安全巩固提升工程，解决2017年预脱贫户饮水难问题。加快推进易地扶贫搬迁，年内易地搬迁贫困群众9000人以上。推进建档立卡贫困户危房改造，解决今年预脱贫户住房难问题。

着力提升贫困村公共服务水平。对贫困群众参加新农合实行缴费补助；推进大病救助，对农村贫困群众住院治疗实行先诊疗后付费；建立贫困户健康档案并完善数据库，做好大病保险、医疗救助、商业健康保险等制度衔接。推进教育脱贫攻坚，加强贫困村学校建设，强化贫困学生资助，年内资助贫困学生37万人次以上。推进文化扶贫，年内建设贫困村村级公共服务中心50个以上。加强农村低保制度和扶贫开发政策衔接，提高农村低保标准。

切实增强脱贫攻坚实效。严格一把手脱贫攻坚责任制，坚持实行督查、通报、协调三项制度，落实脱贫摘帽退出机制、激励约束机制。引导贫困群众自力更生、勤劳致富，提升自身“造血”能力。整合社会扶贫资源，鼓励引导企业及社会爱心人士参与扶贫工作。加大后续扶持力度，巩固现有脱贫成效。

（六）坚持共享发展，着力增进民生福祉

加快教育振兴发展。实施第三期学前教育三年行动计划，学前三年毛入园率达85%以上。促进义务教育均衡发展，义务教育巩固率达95%以上。建设荷城二中、荷城六小、港南一小等学校，建成桂平五中、木乐镇腾飞小学、平南镇东湖小学等学校，基本消除义务教育学校 “大班额”。完成达开高中二期、三期和江南中学改扩建工程，开工建设贵港高中新校区。加快西江职教园区建设，开工建设1-2所大学，圆贵港人民“大学梦”。统筹抓好其他各类教育。

繁荣文化体育事业。基本建成市文化艺术中心，新建村级公共服务中心80个。推进历史文化名城建设，开工修复大南门城楼、翼王亭等古迹遗址。启动《历史名人与贵港》编撰工作。深入开展“和为贵”文化活动，举办音乐会、传统歌戏等各类文艺演出。加强精神文明建设，引导市民自觉践行社会主义核心价值观。开工建设“健康贵港·运动休闲示范区”30个，举办群众广泛参与的篮球、气排球、乒乓球争霸赛和青少年锦标赛。

提高居民健康水平。建成市儿童医院，加快市中心医院建设，完善县级医院、乡镇卫生院、社区卫生服务中心和村医护室医疗设施。提高城乡居民基本医疗保险补助标准和报销比例，年内新农合参保率达95%以上。

优化创业就业环境。落实就业创业扶持政策，加强和完善农民工创业园、创业孵化基地等创业平台建设，引导高校毕业生创业就业，扶持城镇失业人员、农村妇女、农村富余劳动力、退役军人等人群就业，做好农村劳动力转移就业。全面治理拖欠农民工工资违法行为。

完善社会保障体系。实施“全民参保计划”，年内各项社会保险总参保人数730万人次以上。抓好城乡低保、五保供养、医疗救助、临时救助等各项社会救助。加快养老服务业综合改革试验区建设，筹建市老年养护院、港北区老年公寓和一批社区居家养老服务中心、农村幸福院。发展社会福利和慈善事业，加强农村留守儿童、妇女、残疾人和老年人关爱保护工作。加快推进保障性住房建设，建成保障性住房4000套以上。

统筹推进其他事业。加强计生服务管理，控制出生人口性别比。加强城乡社区建设，完成村（社区）“两委”换届。加强应急体系建设，增强预防和处置突发事件能力。开工建设市民中心，健全和完善县级和乡村政务服务中心。完善政府热线受理中心，及时就地解决群众合理诉求。加强安全生产工作，防范重特大事故发生。深化“平安贵港”创建，严密防范和依法惩治违法犯罪活动。加强食品药品监管，确保舌尖上的安全。深入开展民族团结进步创建活动，依法管理宗教事务。强化国防教育，加大国防动员和国防后备力量建设，认真做好优抚安置工作。积极推进外宣工作，提升“荷城”知名度。加强统计工作，强化公积金管理服务和价格监管，推进气象防灾减灾能力建设，重视对台和侨务工作，支持工会、共青团、妇联、科协、残联、社科联和工商联等群团组织参与社会治理，统筹推进机关后勤、绩效、档案、地震等各项工作，密切与中区直部门合作，做好海关、海事、检验检疫、边检、武警、消防、农垦、邮政、通信、水文、石油化工、烟草、电力、金融、盐业、船舶检验、地质勘探、无线电监测等工作。

三、努力提升政府行政执行力

始终坚持党的领导，加强政府自身建设，更加勤勉尽责、务实担当，提升政府行政执行力，推动各项举措落到实处。

加强政府系统党建工作。把全面从严治党要求贯彻到政府自身建设各方面，增强“四个意识”特别是核心意识、看齐意识，在思想上政治上行动上始终同以习近平同志为核心的党中央保持高度一致，不折不扣落实中央、自治区和市委决策部署，确保令行禁止。严格党的组织生活制度，坚持“三会一课”制度、民主生活会和组织生活会制度、谈心谈话制度，对政府系统党员干部进行民主评议。巩固“两学一做”学习教育成果，不断增强政府系统党组织凝聚力战斗力。加强公务员管理，努力打造业务精、能力强、执行好的服务队伍。

着力提高行政效能。秉持“敢干、实干、苦干、善干”作风，坚持不懈推进作风建设，自觉践行市委“五戒五倡”要求，把“马上就办，办就办好”作为工作准则，保持真抓实干的工作状态。坚持实字当头、干字为先，直面问题、说到做到，按照目标明确、措施明确、阶段性目标明确、责任人明确的要求部署工作、推进工作、督查工作和奖惩；坚持问题导向，做到及时主动发现问题、及时主动解决问题、及时主动协调问题、及时主动报告问题，确保工作落到实处。坚持开展“市长服务企业接待日”活动，全面推行“市政府服务接待日”活动和审批职能部门“说不”报告制度。健全督查问责机制，坚决整肃庸政懒政怠政行为。

全面推进依法行政。认真执行市人大及其常委会的决议和决定，全面履行政府职能，规范行政权力，强化严格执法，加快建设法治政府。自觉接受市人大及其常委会的监督，定期向市人大及其常委会报告工作，积极支持市政协履行政治协商、民主监督、参政议政职能，认真听取各民主党派、工商联、无党派人士和各人民团体的意见建议，切实做好人大代表建议和政协提案办理工作。坚持依法决策、民主决策、科学决策，健全决策机制。落实“三重一大”民主决策制度，坚持研究涉及全局的重大事项或作出重大决定及时向市委请示报告，坚持重大事项向市人大常委会报告、向市政协通报。全面推行政务公开政府信息公开，加强电子政务平台建设。建立完善政府新闻发布会制度。

持续加强廉政建设。认真落实中央“八项规定”精神和国务院“约法三章”，严格控制“三公”经费，持之以恒纠正“四风”。坚决反对腐败，加强重点领域、重点项目、重点工作、关键环节的廉政监督，坚决纠正侵害群众利益的不正之风，严厉惩治各种顶风违纪行为，营造风清气正的政治生态。

各位代表！让我们紧密团结在以习近平同志为核心的党中央周围，在自治区党委、政府和市委的正确领导下，凝心聚力，开拓创新，真抓实干，一张蓝图绘到底，咬定青山不放松，不达目的不罢休，为打造“四力”新贵港，加快建成西江流域核心港口和新兴工业城市而努力奋斗！
